# Earth at risk: An urgent call to end the age of destruction and forge a just and sustainable future

**DOI:** 10.1093/pnasnexus/pgae106

**Published:** 2024-04-02

**Authors:** Charles Fletcher, William J Ripple, Thomas Newsome, Phoebe Barnard, Kamanamaikalani Beamer, Aishwarya Behl, Jay Bowen, Michael Cooney, Eileen Crist, Christopher Field, Krista Hiser, David M Karl, David A King, Michael E Mann, Davianna P McGregor, Camilo Mora, Naomi Oreskes, Michael Wilson

**Affiliations:** School of Ocean and Earth Science and Technology, University of Hawai‘i at Mānoa, Honolulu, HI 96822, USA; Department of Forest Ecosystems and Society, Oregon State University, Corvallis, OR 97331, USA; School of Life and Environmental Sciences, University of Sydney, Sydney, NSW 2006, Australia; Center for Environmental Politics and School of Interdisciplinary Arts and Sciences, University of Washington, Seattle, WA 98195, USA; African Climate and Development Initiative and FitzPatrick Institute, University of Cape Town, Cape Town 7700, South Africa; Hui ‘Āina Momona Program, Richardson School of Law, University of Hawai‘i at Mānoa, Honolulu, HI 96822, USA; Hawai‘inuiākea School of Hawaiian Knowledge, Kamakakūokalani Center for Hawaiian Studies, University of Hawai‘i at Mānoa, Honolulu, HI 96822, USA; School of Ocean and Earth Science and Technology, University of Hawai‘i at Mānoa, Honolulu, HI 96822, USA; Institute of American Indian Arts, Santa Fe, NM 87508, USA; Upper Skagit Tribe, Sedro Woolley, WA 98284, USA; School of Ocean and Earth Science and Technology, Hawai‘i Natural Energy Institute, University of Hawai‘i at Mānoa, Honolulu, HI 96822, USA; Department of Science Technology and Society, Virginia Tech, Blacksburg, VA 24060, USA; Doerr School for Sustainability, Stanford Woods Institute for the Environment, Stanford University, Stanford, CA 94305, USA; Department of Languages, Linguistics, and Literature, Kapi‘olani Community College, Honolulu, HI 96816, USA; Global Council for Science and the Environment, Washington, DC 20006, USA; Department of Oceanography, School of Ocean and Earth Science and Technology, Honolulu, HI 96822, USA; Daniel K. Inouye Center for Microbial Oceanography, Research and Education, University of Hawai‘i at Mānoa, Honolulu, HI 96822, USA; Department of Chemistry, University of Cambridge, Cambridge CB2 1DQ, UK; Department of Earth and Environmental Science, University of Pennsylvania, Philadelphia, PA 19104, USA; Department of Ethnic Studies, Center for Oral History, University of Hawai‘i at Mānoa, Honolulu, HI 96822, USA; Department of Geography and Environment, University of Hawai‘i at Mānoa, Honolulu, HI 96822, USA; Department of the History of Science, Harvard University, Cambridge, MA 02138, USA; Associate Justice, Hawaii Supreme Court (retired), Honolulu, HI 96813, USA

**Keywords:** environmental policy, global economics, climate change, biodiversity loss, socioeconomic inequality

## Abstract

Human development has ushered in an era of converging crises: climate change, ecological destruction, disease, pollution, and socioeconomic inequality. This review synthesizes the breadth of these interwoven emergencies and underscores the urgent need for comprehensive, integrated action. Propelled by imperialism, extractive capitalism, and a surging population, we are speeding past Earth's material limits, destroying critical ecosystems, and triggering irreversible changes in biophysical systems that underpin the Holocene climatic stability which fostered human civilization. The consequences of these actions are disproportionately borne by vulnerable populations, further entrenching global inequities. Marine and terrestrial biomes face critical tipping points, while escalating challenges to food and water access foreshadow a bleak outlook for global security. Against this backdrop of Earth at risk, we call for a global response centered on urgent decarbonization, fostering reciprocity with nature, and implementing regenerative practices in natural resource management. We call for the elimination of detrimental subsidies, promotion of equitable human development, and transformative financial support for lower income nations. A critical paradigm shift must occur that replaces exploitative, wealth-oriented capitalism with an economic model that prioritizes sustainability, resilience, and justice. We advocate a global cultural shift that elevates kinship with nature and communal well-being, underpinned by the recognition of Earth’s finite resources and the interconnectedness of its inhabitants. The imperative is clear: to navigate away from this precipice, we must collectively harness political will, economic resources, and societal values to steer toward a future where human progress does not come at the cost of ecological integrity and social equity.

## Climate change and global sustainability

It is unequivocal that human influence has warmed the atmosphere (1) and the climate crisis is now well underway. Global greenhouse gas (GHG) emissions set a new record in 2023 (2), rising an estimated 1.1%, the third annual increase in a row since the COVID-19 recession. With a record 1.45 ± 0.12°C of anthropogenic global heating reached in 2023 (3), we already see nearly one-third of the world population exposed to deadly heat waves (4), a 9-fold increase in large North American wildfires (5), record-setting regional-scale megadrought (6), the Antarctic ice sheet losing nearly 75% more ice between 2011 and 2020 than it did for the period 2001 and 2010 (7), animal and plant extinctions projected to increase 2- to 5-fold in coming decades (8), deepening genetic diversity loss (9), and a weakened global ecosystem (10) pushed to its breaking point (11).

Scientists suspect the last several years have been warmer than any point in more than 125,000 years (12). Yet demand for oil climbed to over 100 million barrels per day in 2023, the highest in history (13). Despite decades of global investment in clean energy (14), fossil fuels still provide over 80% of global energy use (15), a figure that has not changed for decades. In the absence of climate action, our world is on course (16) to heat a blistering 3°C, perhaps more (17), potentially displacing one-third of humanity (18).

One study (19) suggests that ∼9% of people (>600 million) already live outside the human climate “niche.” Another concludes that, compared with people born in 1960, children born today will experience 7.5 times as many heatwaves, 3.6 times as many droughts, 3 times as many crop failures, 2.8 times as many river floods, and 2 times as many wildfires (20). Studies (21) forecast climate-related extinction of 14–32% of macroscopic species in the next ∼50 years, including 3–6 million animal and plant species, even under intermediate climate change scenarios. With continued warming, the frequency of wildfires will increase over 74% of the global landmass by the end of this century (22). Such assessments are conservative as they are based on projections from climate models that may not capture some important processes through which human-caused heating amplifies persistent weather extremes (23, 24).

Of the 40 leading economies, all of which agreed in the 2015 Paris Climate Accord to take all necessary actions to stop global heating below 1.5°C, not one nation is on track to do what they promised (25). Globally, current climate policies are incompatible with limiting global heating to 1.5°C (26). The remaining budget for a 50% chance of keeping warming to 1.5°C is approximately 250 GtCO_2_ as of January 2023, now equal to around 6 years of current emissions (27). The energy plans of countries responsible for the largest GHG emissions would lead to 460% more coal production, 83% more natural gas, and 29% more oil in 2030 than is compatible with limiting global heating to 1.5°C, and 69% more fossil fuels than is compatible with the riskier 2°C target (28).

The market cost of oil, coal, and natural gas is distorted by subsidies and does not include negative externalities related to pollution, climate change, healthcare, and others (29). Worse, the false promise (30) and widespread allure of unregulated quick fixes, such as “net-zero” contracts that lack monitoring, auditing, and verification, threaten to derail even the best-intentioned commercial and governmental plans for climate stabilization (31). Investigations suggest that the great majority of products transacted on carbon offset markets remove very little GHG from the atmosphere (32), and models indicate that even direct removal of atmospheric CO_2_ does not recover former environmental conditions crucial to food and water security or ecosystem restoration (33).

We do not promote a “doom and gloom” philosophy regarding the future of human civilization. We are optimistic that humanity can correct the unsustainable pathway that we are on. Later in this review, we describe necessary steps in this direction. However, we do take an objective and realistic stand on the issue of sustainability. The realities described here quantify a severe and immediate threat to human health and well-being. They emphasize the imperative for a rapid, sweeping reduction in GHG emissions, and highlight stubborn barriers that impede progress. Developed nations, emerging economies, and commercial entities must invest in rapid decarbonization; correct market distortions favoring fossil fuels; and avoid the spurious trap of false “net-zero” offsets as an excuse to continue polluting the atmosphere.

## Imperialism, overpopulation, and resource extraction

Around the world, a growing number of entities and environmental activists are taking action (34). As of December 2022, there have been 2,180 climate-related legal cases filed in 65 jurisdictions, including international and regional courts, tribunals, quasijudicial bodies, or other adjudicatory bodies. Lawsuits related to climate change have more than doubled over the last 5 years as litigants see courts as a way to enhance (or delay) climate action (35). Children and youth, women's groups, local communities, and Indigenous Peoples, among others, are taking a prominent role in bringing these cases and driving climate change governance reform around the world. This “climate justice movement” seeks to extend the principles of human rights and environmental justice by arguing that future generations have a birthright to a safe climate capable of sustaining genuine human development on a healthy and resilient planet (36).

Yet, for hundreds of years, various manifestations of imperialism, such as slavery, settler colonization, economic and cultural dominance, neocolonialism (37), and the forces of globalization, have promoted a mindset of class privilege and wealth. Motivated by profit, the mechanisms of industrial capitalism have pursued relentless resource depletion achieved by subjugation of local communities, erasure of Indigenous knowledge, and unsustainable plunder of the natural world (38).

Modern imperialism is embodied by industrial capitalism, which prioritizes resource extraction and maximizing profit. This paradigm is deeply embedded in the fabric of global affairs, influencing international trade, political dynamics, and the economic frameworks of nations (39). The persistent reliance on extractive economic practices continues to be a significant obstacle to making critical progress in decarbonization, conserving natural resources, and ensuring social equity. For instance, despite decades of international commitments to end deforestation, around 4.1 M hectares of primary tropical rainforest was lost globally in 2022—an increase of 10% over 2021—producing 2.7 Gt of CO_2_ emissions, equivalent to the annual fossil fuel emissions of India (40). Most modern socioeconomic systems still follow extractive rules of exploitation and trade, and ignore natural rates of resource renewal, failing to consider that the end result is catastrophic (41).

Global population growth amplifies the damage wrought by industrial capitalism. On 15 November 2022, the world's population reached 8 billion people. Human population is expected to increase by nearly 2 billion in the next 30 years, and could peak at nearly 10.4 billion in the mid-2080s (42). Cambridge economist Sir Partha Dasgupta developed a rigorous approach to the question “What is optimal human population?” (43). His theory relates population, consumption, and resource capacity, concluding that an optimal global population lies between 0.5 and 5 billion. This theory implies that Earth is already overpopulated relative to ecological carrying capacity. With every additional person added to the planet, wild habitats are disturbed or destroyed by urbanism, agricultural activities, and resource consumption, with humanity demanding more than what the biosphere can sustainably provide.

Dasgupta highlights the critical connection between our economies, livelihoods, and well-being with the Earth’s resources. He argues that current global demand for natural resources surpasses its capacity to supply, driven by factors like population growth and consumption patterns. This overuse threatens biodiversity and ecosystem services. To safeguard our prosperity and the environment, we must rethink our approach to economic success. Key recommendations include increasing nature's capacity and ensuring our demands on nature stay within sustainable limits. This involves investing in natural capital, revising economic metrics, transforming institutions (especially finance and education), and empowering citizens. Legitimate sustainability is vital for achieving a long-term balance between population, economic growth, and the environment. Future generations’ well-being hinges on how we manage economic, social, and natural resources today. Urgent action is required to address these interconnected challenges.

Given the current state of the ecosphere, a 25% increase in population and projected doubling of economic activity by 2050 (44) may drive major ecological regime shifts (i.e. forest to savannah, savannah to desert, thawing tundra, and others) well before 2080. Nature may impose its own population correction before standard projections are realized (45). Actions to slow and reverse population growth are critical (46). These include empowering women, investing in girls’ education, strengthening healthcare systems, and implementing social welfare programs that create job opportunities, reduce poverty, and improve living standards.

Human population growth, increased economic demands, rising heat, and extreme weather events put pressures on ecosystems and landscapes to supply food and maintain services such as clean water. Studies show that ecosystems threatened by sudden regime shifts are at greater risk of collapse than previously thought (47). Researchers warn that more than a fifth of ecosystems worldwide, including the Amazon rainforest, are at risk of a catastrophic breakdown within a human lifetime.

The United Nations’ Sustainable Development Goals (SDGs), a suite of 17 objectives with 169 targets established in 2015 for achievement by 2030, face a grim forecast: current trends suggest none of the goals and merely 12% of the targets may be realized (48). This shortfall underscores the urgent need to dismantle the entrenched model of resource extraction and wealth concentration, advocating for a paradigm shift toward genuine sustainability and resource regeneration. Such a transformation is imperative to reverse the tide of biodiversity loss due to overconsumption and to reinstate the security of food and water supplies, which are foundational for the survival of global populations.

## Global economics and values

**Fig. 1.**
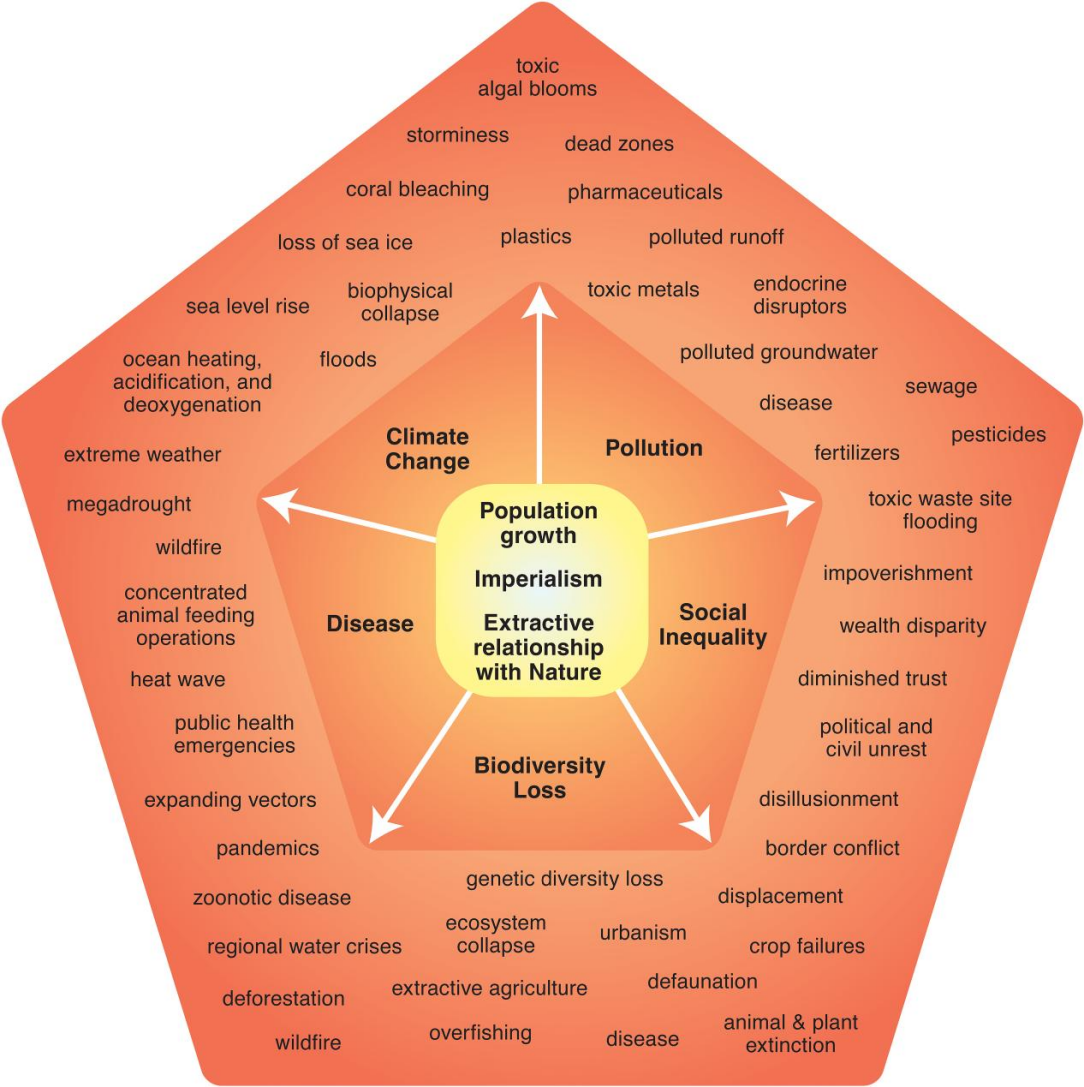
Global population growth, imperialism, and an economic model based on extractive rules of exploitation and trade that ignores natural rates of resource renewal, set the stage for a convergence of several worldwide trends that threaten safe and sustainable human development: accelerating impacts from climate change, pollution, social inequality, biodiversity loss, and disease.

Convergence of worldwide trends threatens safe and sustainable human development: accelerating impacts from climate change (49), biodiversity loss (50) caused by unsustainable consumption (51), extractive agriculture, natural resource exploitation (52) and limitations, emergent disease (53), pervasive pollution (54), and socioeconomic injustice (55). To secure a safe future for humanity, global economics and values must protect the well-being of the natural world. This requires understanding the impacts, intersections and feedbacks of these global emergencies, as well as solutions to ensure a livable planet (56). These emergencies, promulgated by extractive policies (57), human population growth, and modern imperialism (58), overlap in ways that amplify negative outcomes (Fig. 1). If successive governments treat these issues in isolation, hesitate, or formulate shallow responses, the fallout may be catastrophic. Without immediate action, we risk entering a malignant era of global distress and suffering characterized by disease, thirst and hunger, impoverishment, and political instability.

**Fig. 2.**
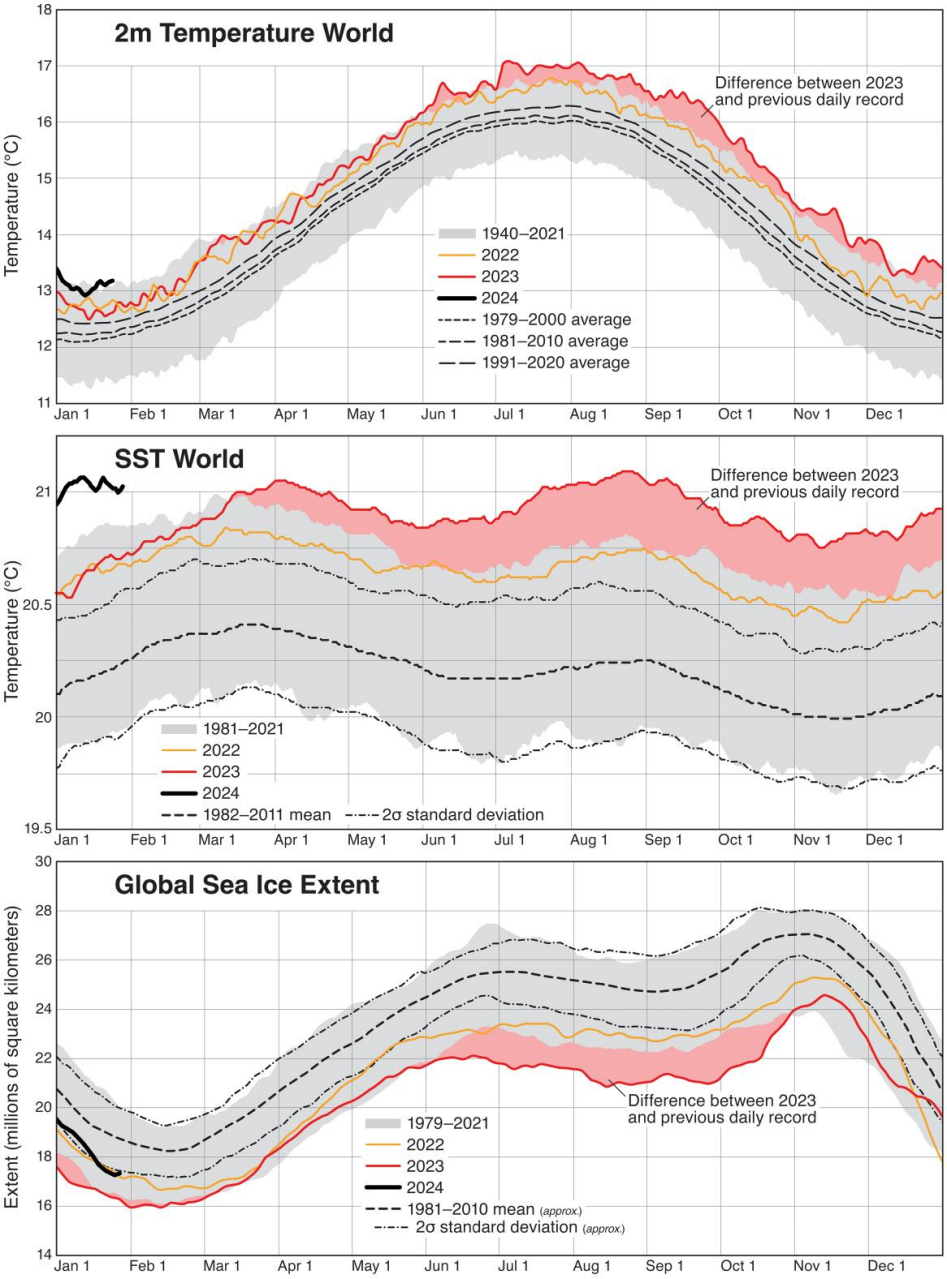
In 2023, astonishing new records were set in 2 m surface temperature, sea surface temperature (SST), and global sea ice extent (2 m Temperature World, and SST World after Climate Reanalyzer, Climate Change Institute, University of Maine, https://climatereanalyzer.org/; Global Sea Ice Extent after https://zacklabe.com/global-sea-ice-extent-conc/).

The cocoon of wealth enjoyed by developed nations belies the suffering and misery many low latitude and semiarid communities already endure in tenuous heat and drought conditions. Consider the Northern Hemisphere summer of 2023. Over 80% of the global population experienced climate change-driven heat in the month of July (59) (Fig. 2). It featured 7 consecutive months of record-shattering global temperature driven by a combination of a moderately strong El Niño and a decrease of Earth's albedo (equivalent to an increase of atmospheric CO_2_ from 420 to 530 ppm) (60). Extreme heatwaves swept many parts of the world. Sea surface temperatures leapt to record highs. Antarctic sea ice was far below average. Record wildfires burned for months destroying tens of millions of acres and produced continental-scale public health crises in air quality, and tens of thousands of temperature records around the world were broken. Without human-induced climate change these events would have been extremely rare (61).

It is past time to build a new era of reciprocity with nature that redefines natural resource economics. The ecological contributions of Indigenous Peoples through their governance institutions and practices are gaining recognition and interest. Indigenous systems of land management encompass a holistic approach that values sacred, ethical, and reciprocal relationships with nature, integrating traditional knowledge and stewardship principles to sustainably manage land and water resources. Indigenous land management challenges conventional power structures and introduces innovative solutions to environmental issues, especially in the context of climate change.

Indigenous Peoples exercise traditional rights over a quarter of Earth's surface, overlapping with a third of intact forests and intersecting about 40% of all terrestrial protected areas and ecologically intact landscapes. These lands typically have reduced deforestation, degradation, and carbon emissions, compared with nonprotected areas and protected areas (62). Beyond western ideas of quarantining land for conservation, Indigenous land management involves a mix of active land management, biomimicry, and conservation to maximize nutrition, food and water security, carbon sequestration, biodiversity, and ecosystem restoration (63). These qualities offer beneficial feedbacks that increase human health and resiliency, build social equity, and provide for the needs of future generations.

We suggest that an Indigenous worldview, that of kinship with nature, should define sustainable practices. Laws that establish legal rights for nature have reached a critical point at which they may either be normalized or marginalized (64); this progress must be sustained. For instance, Māori in New Zealand have successfully asserted sovereignty to grant legal personhood to the Whanganui River and Te Urewera National Park. This reflects Māori worldviews and recognizes their governance, allowing “nature” to have a legal voice. In the US, the Menominee Forest Management Reserve, recognized as a best practice, is driven by the Menominee vision and worldviews. It operates under the recognition of Menominee sovereignty and decision-making authority.

Nations must build on these regenerative practices by eliminating environmentally harmful subsidies (65), and restricting trade that generates pollution and unsustainable consumption. Studies (66) indicate the global economy must achieve absolute decoupling (in which resource impacts decline in absolute terms) (67) if we are to eliminate “ecological overshoot”^a^ (68).

**Fig. 3.**
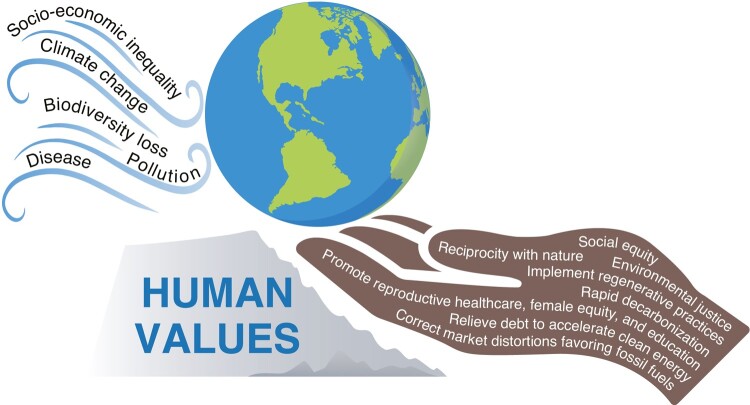
The stability of human communities and natural ecosystems is at risk under the shocks and stresses of five planetary emergencies: socioeconomic inequality, climate change, biodiversity loss, pollution, and disease. Unless human values shift dramatically and soon, the resulting damage to the natural world will likely be catastrophic, with long-lasting consequences for species and ecosystems, and devastating upheavals for humanity. A systemic change in human values is needed that focuses on Earth-centered governance, and entails a transition in collective values, behaviors, and institutional practices to prioritize long-term ecological health and social well-being over immediate gains.

In the words of coauthor Jay Bowen, Upper Skagit Elder, “We are all Indigenous to this Earth. We are one family.” The authors of this review believe that humanity stands at an inflection point in human history that will determine many characteristics of future life on Earth (Fig. 3). Continued failure to integrate these problems in climate resilient development and regenerative practices risks the stability of human communities and natural systems. Heads of state must recognize the existence of a global emergency (56), treat these crises as intertwined issues, and apply the considerable power of the economy toward restoring a livable planet and an equitable and just socioeconomic system before climate instability and ecological regime shift are beyond our control. Later in this paper, we offer specific suggestions for implementing these changes.

## Climate realities and the road to action

In April 2023, CO_2_ levels measured at Mauna Loa Observatory in Hawai‘i reached an annual peak of 424.8 ppm, more than 50% greater than the preindustrial level of 278 ppm. In the first decade of measurement at Mauna Loa (1959–1968), the average annual growth rate was 0.8 ppm per year. The average annual growth rate over the most recent decade (2014–2023) was 3 times that amount, 2.4 ppm per year, the fastest sustained rate of increase in 65 years of monitoring (69).

More than half of all industrial CO_2_ emissions have occurred since 1988 and 40% of the CO_2_ we emit today will still be in the atmosphere in 100 years, about 20% will still be there in about 1,000 years (70). The last time CO_2_ levels were this high was the Pliocene Climatic Optimum, 4.4 milion years ago, when Earth's climate was radically different; global temperature was 2–3°C hotter, beech trees grew near the South Pole, there was no Greenland ice sheet, no West Antarctic ice sheet, and global sea level was as much as 25 m higher than today (71).

Atmospheric methane (CH_4_) growth has surged since 2020. Averaged over 2 decades, the global heating potential of CH_4_ is 80 times greater than CO_2_. The largest sources of atmospheric CH_4_ are wetlands, freshwater areas, agriculture, fossil fuel extraction, landfills, and fires. In 2023, atmospheric CH_4_ exceeded 1,919 ppb, on track to triple the preindustrial level of 700 ppb by 2030. Carbon isotopic signatures reveal microbial decomposition of organic matter as the major source of CH_4_ emissions, indicating that natural CH_4_-producing processes are being amplified by climate change itself (72). Is this a sign that global heating is shifting beyond our control?

Under an intermediate scenario (SSP2-4.5), GHG emissions are very likely to lead to heating of 1.2–1.8°C in the near term (2021–2040), 1.6–2.5°C in the midterm (2041–2060), and 2.1–3.5°C in the long term (2081–2100) (73). As of November 2023, 145 countries had announced or are considering net-zero targets, covering close to 90% of global emissions (74). Among these are China, EU, USA, and India, who jointly represent more than half of global GHG emissions. However, net-zero evaluations for G20 countries and selected other countries as of November 2023 show that most net-zero targets are formulated vaguely and do not yet conform with good practices.

Even as the vast majority of countries pledged to slash their climate emissions, their own plans and projections put them on track to extract more than twice the level of fossil fuels by 2030 than would be consistent with limiting heating to 1.5°C, and nearly 70% more than would be consistent with 2°C of heating (28). The world has a 67% chance of limiting warming to 2.9°C if countries stick to the nationally determined contributions (NDCs) made under the 2015 Paris agreement (26). Emission cuts of 14 GtCO_2_ or 28% are needed by 2030 to keep within 2°C of warming. A reduction of more than 40% or 22 GtCO_2_ is needed for the 1.5°C threshold to be realistic.

The world now only has a 14% chance of limiting warming to the 1.5°C goal, even if countries honor all NDCs. Limiting warming to 1.5°C would require global emission reduction of 8.7% per year. Even with COVID-19 lockdowns limiting manufacturing, ground and air transportation, and other economic activities during 2020, emissions dropped by only 4.7% (26).

Many countries’ net-zero pledges “are not currently considered credible” (26). No G20 country is reducing emissions at a pace consistent with their net-zero targets. The lifetime emissions of current and planned oil and gas fields and coal mines is 3 and a half times greater than the carbon budget needed to hold temperature increase to 1.5°C. It would exhaust almost all the budget needed for 2°C.

Under current national climate plans, emissions are expected to rise 9% above 2010 levels by the end of this decade even if NDCs are fully implemented. GHG emissions would fall to 2% below 2019 levels by 2030. Although these numbers suggest the world will see emissions peak this decade, that's still far short of the 43% reduction against 2019 levels that the Intergovernmental Panel on Climate Change (IPCC) says is needed to stay within the 1.5°C target envisioned by the Paris Agreement (26).

Emission reductions of 43% are needed by 2030 to keep 1.5°C in play. But since the 26th Conference of Parties (COP) in 2021, nations have shaved just 1% off their projected emissions for 2030, and COP 28 in 2023 ended with no increase in ambition. Seventy-five percent of nations that have set targets to limit GHG emissions have enshrined them in law or policy documents, but the plans needed to implement those pledges are lacking in almost all cases (74), and policies based on “net-zero” actions no longer have credibility. Current pledges would lead to long-term global heating of 2.4–2.6°C, but on-the-ground policies put the world on track for heating approximately 3°C above preindustrial levels. Avoiding dangerous levels of heating requires systemic transformation to energy, waste, transportation, agriculture, and industry.

Climate indicators show that global heating reached 1.14°C averaged over the past decade, 1.26°C in 2022, and 1.45 ± 0.12°C over the 12-month period of 2023. In 2023, some 7.3 billion people worldwide were exposed, for at least 10 days, to temperatures influenced by global warming, with one-quarter of people facing dangerous levels of extreme heat. Heating is increasing at an unprecedented rate of over 0.2°C per decade (perhaps faster) caused by a combination of annual GHG emissions at an all-time high of 54 ± 5.3 GtCO_2_e over the last decade, and reductions in the strength of aerosol cooling (17). The Northern Hemisphere summer of 2023 revealed a shift in climate indicators marking a new level of intensity. “There has never been a summer like this in recorded history: shocking ocean heat, deadly land heat, unprecedented fires and smoke, sea ice melting faster than we’ve ever seen or thought possible (75).”

## Climate outlook

Planned cuts in global emissions are inadequate for protecting human security and Earth's remaining biodiversity. Under implemented national policies alone, dangerous heating is only avoidable with a massive rollout of GHG removal technologies and large-scale ecosystem restoration that is nowhere in evidence today. For instance, even the planned investment of $3.5B to develop four “direct air capture” hubs under the 2022 US Bipartisan Infrastructure Law will only remove the equivalent of 13 min of global emissions at full annual capacity (30). Planting 8 billion trees, one for every person on Earth, would remove the equivalent of only 43 h of global emissions after the trees reached maturity decades from now, and the change in albedo related to the new ground cover increases the complexity of expected benefits.

The only honest strategy for today is radical, immediate cuts in fossil fuel use. Only after emissions have begun a rapid downward trajectory should investments in carbon removal (the engineering for which has yet to be defined or validated) occur with speed and at scale (76). Even this will be met with ocean outgassing of CO_2_ such that climate recovery will see a long delay (33).

This urgency is underscored by the fact that current emissions are underreported, and decreasing natural carbon storage makes limiting global temperatures even more challenging. Global emissions are as much as 3 times higher than reported (77) with 70% underreporting of energy-related CH_4_ emissions alone (78). In addition, the terrestrial biome, which sequesters about 31% of anthropogenic CO_2_ emissions, has already neared, and in places crossed, a photosynthetic thermal maximum beyond which terrestrial carbon storage will grow increasingly impossible (79). For instance, global carbon loss from tropical forests has doubled in the last 20 years (80), and the Brazilian portion of the Amazon Forest has become a net GHG source (81). Eighty-three percent of tropical forest carbon loss is driven by agriculture, suggesting that strategies to reduce deforestation have failed, and that carbon emissions from forest destruction are undercounted (82).

The United Nations estimates that 1.84 billion people worldwide, or nearly a quarter of humanity, were living under drought in 2022 and 2023, the vast majority in low- and middle-income countries (83). Megadrought projected for the year 2100 could strike up to 50 years earlier according to models (84). Global heating risks food (85) and water (86) availability with human populations in conditions of extreme to exceptional drought (87) doubling by 2100 (88).

Climate change threatens natural ecosystems (89), human security (90), livable conditions for communities (91), and the stability of 1/3 of the human population (18). Under current levels of heating, people are 15 times more likely to die from extreme weather than in years past, and 3.3 billion human lives are “highly vulnerable” to climate change (92). At 2°C heating, up to 3 billion people may suffer chronic water scarcity. Today, 1 in 3 people are exposed to deadly heat stress. This number is projected to increase up to 75% by the end of the century.

By 2050, over 300 million people living on coasts will be exposed to flooding from sea level rise (93). Forced to migrate, the impacts of these displaced communities will ripple through the larger population. Climate change drives the spread of disease in people, crops, domesticated animals, and wildlife. Even if heating is held below 1.6°C, 8% of today's farmland will be unfit to produce food. Declining food production and nutrient losses will result in severe stunting affecting 1 million children in Africa alone and cause 183 million additional people to go hungry by 2050 (92).

## Abrupt change

Earth's biophysical systems are shifting toward instability (94), perhaps irreversibly (95). The IPCC has identified 15 Earth system components with potential for abrupt destabilizing change, including ice, ocean, and air circulation; large ecosystems; and precipitation. These systems are the pillars of life that permit stable plant, animal, and microbial communities, food production, clean water and establish the conditions for safe human development. However, these systems may be characterized by threshold behavior. That is, they appear to remain stable as global temperature rises, but at a certain level of heating, they may “tip” into a fundamentally irreversible new state (96).

As Earth retains heat, ice melt accelerates (97), especially in the Arctic which is heating nearly 4 times faster than the global average (98). Arctic sea ice is declining (99), and the transition from a snow- to rain-dominated Arctic in the summer and autumn may occur as early as 2040, with profound climatic, ecosystem, and socioeconomic impacts (100). The Greenland Ice Sheet is vulnerable to ice loss due to melt-elevation feedback (101), and Greenland is losing ice 7 times faster than in the 1990s (102). Antarctic melting has tripled in the past 5 years (103), and ice shelf collapse may lead to amplified sea level rise (104, 105).

According to one study, if temperatures rise by 1.5°C, the loss of four biophysical systems will become “likely” and loss of an additional six will be “possible.” Loss of 13 biophysical systems will be either “likely” or “possible” if the planet warms by 2.6°C, as expected under current climate policies (94). Emerging changes such as deep ocean heating (106), marine stratification (107), declining marine vertical circulation (108), and sea level rise (109) will continue for centuries even if net-zero emission targets are reached. The Intergovernmental Panel on Climate Change Assessment Report 6, Working Group I (110) projects possibly abrupt and irreversible change in permafrost carbon, West Antarctic ice sheets and shelves, and ocean acidification and deoxygenation. These changes could unleash feedback loops that place climate impacts beyond our control (111).

## Oceans

The world's oceans face irreversible impacts from climate change, with heating, acidification, stratification, and loss of dissolved oxygen posing high costs for marine ecosystems (112). Ocean heating has intensified (113), with the Southern Ocean taking up most of the excess heat generated by anthropogenic activities (114). These changes affect marine species distributions, interactions, abundance, and biomass. Combined with other stressors like pollution, they are putting marine biodiversity and its societal benefits at risk (115).

Amplified by global heating (116), marine biodiversity is being decimated by more than 440,000 industrial fishing vessels around the world that are responsible for 72% of the world's ocean catch. Over 35% of the world’s marine fishery stock is overfished and another 57% is sustainably fished at the maximum level (117). One study showed that more than 90% of the world's marine food supplies are at risk from environmental changes such as rising temperatures and pollution, essential to over 3.2 billion people. Top producers like China, Norway and the United States face the biggest threat (118). Marine heatwaves (119) are increasing with negative impacts on marine organisms and ecosystems. Marine coastal biodiversity is at risk, with over 98% of coral reefs projected to experience bleaching-level thermal stress by 2050 (120).

Relative to the period 1995–2014, global mean sea level is conservatively projected to rise 0.15–0.29 m by 2050, and 0.28–1.01 m by 2100 (109). Higher rise would ensue from disintegration of Antarctic ice shelves and faster-than-projected ice melt from Greenland (121). On multiple occasions over the past 3 million years, when temperatures increased 1–2°C, global sea levels rose at least 6 m above present levels (122). Sea level rise will flood toxic waste sites, cesspools and septic systems, municipal dumps, and polluted groundwater. In many cases, communities of color will be first to experience health impacts (123).

Ocean pollution affects marine species and people who depend on them. Toxic metals, plastics, manufactured chemicals, petroleum, urban and industrial wastes, pesticides, fertilizers, pharmaceutical chemicals, agricultural runoff, and sewage are the most detrimental and persistent pollutants (124). More than 80% of marine pollutants originate from land-based sources, reaching the oceans through rivers, runoff, and atmospheric deposition. Pollution is heaviest in coastal waters, especially in low- and middle-income countries (125).

Toxic metals such as mercury, lead, and cadmium accumulate in marine animals, causing health problems in fish species and disrupting endocrine systems in their human consumers (126). Plastics take hundreds of years to degrade, breaking down into microplastics that are ingested by fish, humans, and other organisms (127). Manufactured chemicals such as polychlorinated biphenyls and dioxins are environmentally persistent toxins that accumulate in the tissues of marine animals, disrupting hormonal systems (128). Urban and agricultural runoff, and sewage contain pathogens (129), heavy metals, and organic compounds that harm marine animals and cause human health problems. Nitrogen pollution also results in toxic algal blooms and oxygen-depleted dead zones (130). The equity and justice implications of this massive problem have been largely overlooked or downplayed (131).

## Terrestrial biome

Tropical forests now emit more carbon than they are able to absorb from the atmosphere as a result of the dual effects of deforestation and land degradation (132). Rich-nation demand (133) for lumber, minerals, beef, and animal feed outside their own borders undermine attempts to mitigate climate change (134). Demand for food, feed, fiber, minerals, and energy is resulting in whole forests being clear-cut. CO_2_ emissions from boreal forest fires have reached a new high, producing nearly 1/4 of the total global CO_2_ emissions from wildfires (135). Only 40% of remaining forests have high ecosystem integrity (136). Forests are degraded (137) by drought, pests, and wildfire related (138) to climate change.

Forest loss sacrifices soil biodiversity and integrity to oxidation, dehydration, and heating, transforming soil into a persistent source of CO_2_ emission (139). Only 2.9% of Earth's land remains ecologically intact (140). Essential ecosystems are disappearing, and many species are at risk of extinction (141). Anthropogenic extinction rates are driving Earth's sixth mass extinction (142). Each year, the world consumes more than 92 Gt of materials—biomass (mostly food), metals, fossil fuels, and minerals. This figure is growing at the rate of 3.2% per year. Resources are being extracted from the planet 3 times faster than in 1970, even though the population has only doubled within that time (143). During the 20th century, this boosted the global economy, but since then resources have become more expensive to extract and the environmental costs harder to ignore.

Both plant and soil carbon storage originate with photosynthesis, which withdraws about 31% of annual anthropogenic CO_2_ emissions (2). However, studies (144) across a range of forest ecosystems have found that heating leads to thermal stress and reduced carbon assimilation. Many ecosystems (80) are already operating at or beyond thermal thresholds for photosynthesis (145). Widespread terrestrial ecological decline has resulted from the combination of climate change, resource extraction, bushmeat hunting, and agricultural and urban development. Since 1970, vertebrate populations have declined 69% (146), and 1 in 4 species are at risk of extinction (147), in part because 75% of the terrestrial environment has been severely altered by human actions.

Agricultural development has further eroded ecosystem health, with over 15 billion trees per year lost since the emergence of agriculture; the global number of trees has fallen by over 45% (148). An estimated 67,340 km^2^ of global forest were lost in 2021 alone, unleashing 3.8 Gt of GHG emissions, roughly 10% of the global average (149). Such losses extend to wetland areas; more than 85% of the wetlands present in 1700 had been lost by 2000, and loss of wetlands is currently 3 times faster than forest loss.

## Food and water security

Increasing human population, and the need to expand food production, were the drivers of the Green Revolution over 50 years ago (150). This increased productivity through selective genetic breeding, monocultures, seed improvement, and the use of chemical fertilizers and pesticides. These steps have not solved the problem of food insecurity which has been aggravated in more vulnerable populations (151). Worldwide, it is estimated that 16,000 children are pushed into hunger every day—a 32% increase from 2022 (152).

Agriculture now uses half of the world's ice- and desert-free land, and causes 78% of global ocean and freshwater eutrophication (153). Pesticide and fertilizer runoff, as well as sewage, find their way to aquatic environments (154) and degrade water quality, while spreading infectious diseases. Humans poison the soil annually with microplastics between 4 and 23 times more than we do the oceans. Microplastics reduce beneficial bacteria concentrations, and can be absorbed by plants, and then passed up the food chain (155).

Industrial farming employs deep plowing that depletes and oxidizes soil, turning acreage into a source of GHG (156). Agriculture is responsible for 70% of global freshwater withdrawals (157). By one estimate (158), 94% of nonhuman mammal biomass is now livestock, and 71% of bird biomass is poultry livestock. 50% of all agricultural expansion has come at the expense of forests. In 2022, the rate of global deforestation was the equivalent of 11 soccer/football fields per minute (40), predominantly for cattle ranching and grain animal feed crops (such as soy) for export.

Today, agriculture uses half of all habitable land (159), and either through grazing or growing animal feed, 77% of that is dedicated to livestock (153). Animal agriculture is expanding. From 1998 to 2018 global meat consumption increased 58%. Cattle and the grain they eat use 1/3 of all available land surface, 1/3 of global grain production, and 16% of all available freshwater. Yet cattle agriculture only generates 18% of food calories and 27% of protein (153). The production of fertilizer for feed crops emits 41 MtCO_2_/yr. The combination of emissions from manufacturing, transporting, and applying synthetic fertilizer on the land (which releases the potent GHG N_2_O) today likely outpaces the emissions of the commercial aviation industry. These fertilizer-related GHG emissions are projected to grow. Additionally, livestock feed demands a minimum of 80% of global soybean crop and over 50% of global corn crop. Thirty-five to 40% of yearly anthropogenic CH_4_ emissions are a result of domestic livestock production due to enteric fermentation and manure (160).

Under a range of GHG emission pathways, cropland exposure to drought and heat-wave events will increase by a factor of 10 in the midterm and a factor of 20–30 in the long term on all continents, especially Asia and Africa (161). Harvest failures across major crop-producing regions are a threat to global food security. Jet stream changes are projected to increase synchronous crop failure and lower crop yields in multiple agricultural regions around the world (162). Crop failure due to drought, flood, or extreme weather (163) events increases disproportionately between 1.5 and 2°C of global heating (164). For maize, risks of multiple breadbasket failures increase from 6– 40% at 1.5°C to 54% at 2°C. In relative terms, the highest climate risk increases, between 1.5 and 2°C heating, is for wheat (40%), followed by maize (35%) and soybean (23%). Limiting global heating to 1.5°C would reduce the risk of simultaneous crop failure for maize, wheat, and soybean by 26%, 28%, and 19%, respectively (164).

Demand for wheat is projected to increase 60% by 2050. Yet, rising CO_2_ depletes the nutrient and protein content of wheat, and with drought, fire, and flood, leads to a 15% decline in projected wheat yield by midcentury (165). Increased levels of CO_2_ are decreasing the amount of protein, iron, zinc, and B vitamins in rice with potential adverse health consequences for a global population of approximately 600 million (166). Harvests of staple cereal crops, such as rice and maize, could decrease by 20–40% as a function of heightened surface temperatures in tropical and subtropical regions by 2100 (167). This will exacerbate existing food security issues, as 1 billion people are currently classified as food insecure (168).

Worldwide, fungal infections cause growers to lose 10–23% of their crops each year, and an additional 10–20% is lost following harvest. Global heating is driving a poleward migration of fungal infections, meaning more countries will see fungal infections damaging harvests. Growers have reported wheat stem rust infections, usually tropical, in Ireland and England. Experts (169) also warn that fungi tolerance to higher temperatures could increase the likelihood of soil-dwelling pathogens to infect animals or humans. Across the five most important calorie crops of rice, wheat, maize (corn), soybeans, and potatoes, fungal infections already cause losses equal to provisions for 600 million to 4 billion people. Without major and rapid policy changes, food productivity in 2050 could be reduced to 1980 yield levels because new technologies will be unable to mitigate climate change in major growing regions (170).

Clean water security is a critical issue (171). Research shows that groundwater levels are rapidly declining, especially in dry regions with extensive croplands, and has accelerated over the past four decades in 30% of the world's regional aquifers (172). The Southern Hemisphere has experienced a 20% drop in water availability over the past two decades (173). Approximately 3.6 billion people, or 47% of the global population, suffer water scarcity at least 1 month each year (174). Global water security is an urgent concern due to the increasing imbalance between the finite supply of freshwater and the escalating demand driven by population growth, economic development, and agricultural needs. Climate change compounds the crisis by altering precipitation patterns, causing droughts, and depleting glaciers—key freshwater sources. Contamination from industrial, agricultural, and residential waste further restricts the amount of clean water available. This scarcity threatens human health, food production, and ecosystem stability, leading to conflicts and displacements. Addressing this problem requires global cooperation for sustainable management, technological innovation for conservation and purification, and policies that prioritize equitable access to clean water (174).

## Heat

The impact of heat on food production is disproportionately severe in low-income communities. Workers in agriculture, construction, and other outdoor sectors often work in conditions that can lead to heat stress or heatstroke. Food production, too, is critically affected as extreme heat can reduce crop yields, increase irrigation needs, and lead to soil degradation. These communities have less access to heat-protection technologies such as air-conditioned spaces, efficient irrigation systems, or heat-resistant crop varieties. Consequently, their economic stability and food security are more vulnerable to climate-induced temperature increases, exacerbating existing inequalities and pushing these populations further into poverty.

In 2022, global heat stress caused the loss of 490 billion potential labor hours, 143 h per person, a 42% increase from the 1991 to 2000 average (175). The loss of labor due to heat exposure resulted in a $863 billion loss of “potential income” and wiped out the equivalent of 4% of Africa's GDP. The agriculture sector was hardest hit, accounting for 82% of losses in least developed countries. The global land area affected by at least 1 month of extreme drought per year increased from 18% averaged over the decade 1951–1960 to 47% in the decade 2013–2022. Because of heat stress, under a 2°C warming scenario, 525 million additional people will experience food insecurity by midcentury, compared to the period 1995–2014, and the number of heat-related deaths each year will increase by 370%. Older people and infants now are exposed to twice the number of heat-wave days annually as they were averaged over the period 1986–2005.

Heat-related deaths of people older than 65 have increased by 85% since the 1990s (175). Even under moderate warming, heat and drought levels in Europe that were virtually impossible 20 years ago reach 1-in-10 likelihoods as early as the 2030s (84). Averaged over the period 2050–2074, projections for two successive years of single or compound end-of-century extremes, unprecedented to date, exceed 1-in-10 likelihoods; while Europe-wide 5-year megadroughts become plausible. Whole decades of end-of-century heat stress could start by 2040, by 2020 for drought, and with a warm North Atlantic, end-of-century decades starting as early as 2030 become twice as likely.

For thousands of years, fundamental limits on food and water security meant that human communities have concentrated under a narrow range of climate variables characterized by mean annual temperatures (MATs) around 13°C (18). With continued GHG emissions, global heating of 3°C is projected to drive a MAT >29°C across 19% of the planet's land surface and displace one-third of the human population. Today, this MAT accounts for only 0.8% percent of Earth's land surface, mostly concentrated in the deep Sahara.

Model projections indicate that in the Middle East and North Africa, continued emissions will cause the emergence of unprecedented super- and ultraextreme heat-wave conditions (176). These events involve excessively warm temperatures (56°C and higher) and will be of extended duration (several weeks), quickly becoming life-threatening for humans (177). Researchers found that by 2100, under current levels of GHG emissions, 3 of 4 people in the world will be exposed to deadly heat conditions every year, with a higher occurrence of these conditions in intertropical areas (2). Coupled with significant socioeconomic differences within countries, heat waves intensify global disparities in health, especially given the depleted resources for several of these regions to respond to accelerated heating. In the last decade, there has been >2,300% increase in the loss of human life from heat waves as a result of about 1°C heating. On our current pathway, the global health and socioeconomic risks of continued heating are catastrophic.

The distribution of these conditions is unequal, and people and communities subjected to the loss of security are powerless to respond. The impacts of this inequity may cause regionally existential deterioration and suffering. As temperatures rise, death rates increase most among the poorest populations (178). By 2099, under a scenario of continued high emissions growth, climate change increases death rates in low-income countries by over 106 deaths per 100,000, while high-income countries are projected to see death rates decrease by 25 deaths per 100,000, while spending significantly to prevent more deaths. Overall, today's rich countries pay nearly 3 times more than poor countries to adapt to rising temperatures and prevent additional deaths. When it comes to cutting emissions, the social and economic burden of inaction is predominantly carried by the poorest and most vulnerable in human society, including Indigenous and local communities, concentrated in developing countries.

## Illness and disease

As the planet heats up, infectious diseases once confined to tropical regions are expanding their range. The World Health Organization estimates that by the end of this decade the climate impact on health will cost between $2 billion and $4 billion per year (179). Between 2030 and 2050, climate change is expected to cause approximately 250,000 additional deaths per year from, for example, undernutrition, malaria, cholera, diarrhea, and heat stress alone. This does not include massive climate burdens on agriculture, water, and sanitation, which also shape public health.

In July 2023, for the first time in 20 years, the United States experienced locally acquired malaria infections. Six cases were confirmed in Florida and one in Texas, none related to international travel (180). In Seattle, cases of West Nile disease were reported for the first time. Over half of the infectious diseases confronted by humanity have been aggravated by climatic hazards at some point (181). All communities are vulnerable to climate change impacts; however, children, elders, the sick, and the poor face the greatest risks (182). People with cardiovascular and/or respiratory chronic illnesses are particularly vulnerable to high temperatures (183). Air pollution from GHG emissions leads to increased health complications such as asthma and allergies. The impacts of climate change disproportionately affect vulnerable communities, including low-income regions and communities of color which have been disempowered by a history of colonialism, racism, oppression, and injustice. Extreme weather events further exacerbate the situation, driving animals and people together in unsanitary conditions and disrupting essential services like healthcare and clean water supplies.

Approximately 17% of diseases are spread by animal vectors causing over 700,000 deaths annually. Concentrated animal farming operations are breeding grounds for virulent pathogens (184), and over 15,000 new cases of mammals transmitting viruses to other mammals could occur in the next 50 years due to climate change (185). Smaller species like bats, rats, and other rodents are thriving in human-populated areas, contributing to the spread of diseases through their interactions. Biodiversity loss and deforestation are directly linked to the rise of infectious diseases, with 1/3 of zoonotic diseases attributed to these factors. Some 60% of known pathogens, and 3 out of every 4 new or emergent infectious diseases are zoonotic (186), and roughly 1/3 of those are attributed to deforestation and habitat loss (187). A new disease surfaces 5 times a year, and future global heating and precipitation changes will further expand habitats for pathogens and vectors, proliferating dengue fever, cholera, malaria, diarrhea, and other diseases (188).

Climate change intensifies the spread of infectious diseases, particularly in low-income communities, by expanding the habitats of disease vectors such as mosquitoes and ticks. Warmer temperatures and altered rainfall patterns increase the incidence and geographic range of vector-borne diseases like malaria and dengue fever. Flooding and extreme weather events, more common as the climate changes, can lead to waterborne diseases due to the contamination of freshwater supplies. Low-income areas often have insufficient healthcare infrastructure, making them more vulnerable to these outbreaks. Additionally, malnutrition from climate-induced food scarcity can weaken immune systems, further raising the susceptibility to infections. Thus, climate change magnifies health disparities, with low-income communities facing disproportionately high risks of disease.

## Economic inequality, ecological destruction, and global security

A grossly unequal distribution of wealth couples with the increasing consumption patterns of a rising global middle class (189) to amplify ecological destruction. The poorest half of the global population owns barely 2% of total global wealth, while the richest 10% owns 76% of all wealth (190). The poorest 50% of the global population contribute just 10% of emissions, while the richest 10% emit more than 50% total carbon emissions (191). Climate change, economic inequality, and rising consumption levels intertwine to amplify ecological destruction.

Climate change, driven by carbon emissions, often stems from industrial activities catering to increased consumption, particularly in wealthier nations. This consumption depletes natural resources and exacerbates pollution and habitat loss. Economic inequality compounds these issues, as poorer communities lack the resources to adapt to environmental changes or invest in sustainable practices. Consequently, low-income communities bear the brunt of ecological degradation, such as soil erosion, water scarcity, and biodiversity loss, while their limited economic means prevent effective response or recovery. This cycle of consumption, inequality, and environmental impact creates a feedback loop, perpetuating and intensifying ecological damage globally.

Fifty years ago, underdevelopment and scarcity were drivers of unsustainable resource use, but today these roots have morphed into overdevelopment, affluence, and privilege driving unsustainable wealth accumulation and aggregate consumption. At present, not a single country delivers what its citizens need without transgressing planetary boundaries of long-term sustainability (192). Modern imperialism amplifies these inequalities through economic exploitation, wealth accumulation, political interference, cultural dominance, and other methods that leverage colonial power structures. Recognizing and addressing neocolonial practices is crucial for promoting equitable and sustainable development and respecting the sovereignty and self-determination of nations (193).

The use of natural materials and their benefits are unevenly distributed across the globe. Overconsumption is closely linked to wealth and income disparities with large amounts of money concentrated in a few rich countries, largely in the Northern Hemisphere (194). For example, environmental stresses and shocks related to natural resource extraction and use are outsourced to countries and regions outside the European Union, while more than 85% of the economic benefits stay within member countries (195).

Global inequality results in fragile regions where intensified conflict over scarce resources allows malevolent actors to thrive (196). One study (197) found strong causal evidence linking climatic events to human conflict across all major regions of the world: for each 1 SD (1σ) change in climate toward warmer temperatures or more extreme rainfall, data show that the frequency of interpersonal violence rises 4% and the frequency of intergroup conflict rises 14%. Temperatures across the developed world are expected to warm 2σ to 4σ by 2050. Hence, amplified rates of human conflict could represent a large and critical impact of anthropogenic climate change.

Over the next 3 decades, even under best-case scenarios of low heating, national, and global security face severe risks in every region of the world. Higher levels of heating will pose catastrophic, and likely irreversible, global security risks over the course of the 21st century. A world where global mean surface temperature has increased 3°C will be characterized by widespread and intense heat stress, extreme weather events, ruptured and unproductive marine and terrestrial ecosystems, broken food systems, disease and morbidity, intense decadal megadrought, freshwater scarcity, catastrophic sea level rise, and large numbers of migrant populations. By 2050, under these malignant conditions, up to 1.2 billion humans could be displaced by climate change (198). These intensifying crises now threaten the very fabric of our global socioeconomic system. Immediate action is imperative to avert a collapse that endangers societal structures worldwide.

## Climate purgatory

Although the global condition is bleak, after 200 years of fossil fuel expansion, we are at a turning point in the energy system. The clean-energy revolution is underway. Global sales of vehicles powered by fossil fuels peaked in 2017 (199), and in 2023 electric vehicle sales grew by 55%, reaching a record high of more than 10 million. For the first time ever, announced manufacturing capacity for electric vehicle batteries is now sufficient to fulfill expected demand requirements by 2030 (200).

Renewable energy installations jumped nearly 50% in 2023, the most rapid growth rate in two decades (200). After remaining flat for several years, global clean energy spending is increasing. Last year, renewables made up about 30% of total electricity generation, up from 25% in 2018. Global investment in the energy transition totaled $1.77 trillion in 2023, an increase of 17% from the prior year. Solar energy is expected to become the cheapest form of energy in many places by 2030 and major global powers are investing in infrastructure for energy transformation.

However, increasing global energy consumption offsets these gains in renewable energy. Because of rising power needs in developing nations due to population growth and industrialization, ongoing electrification of the transport and building sectors, and other areas of energy expansion, the International Energy Agency (IEA) projects increasing growth of energy demand, rising at an annual average rate of 3.4% in 2024–2026. Although the expansion of clean-energy sources is set to meet this demand growth, decoupling energy consumption and CO_2_ production, the separation is not nearly wide enough to meet Paris Agreement Goals for stopping global heating.

Countries and companies are taking steps to address climate change while simultaneously making choices that undermine these efforts. This paradox places us in a state of climate purgatory. The IEA predicts (200) a peak in fossil fuel demand by 2030, but reports show governments planning to increase coal, oil, and gas production well beyond climate commitments. This math does not align with the 1.5°C or even the 2°C warming targets. Many experts consider these targets nearly impossible due to the global reluctance to urgently phase out fossil fuels. In this climate purgatory, we are at a critical juncture, where urgent, transformative action is required to reconcile our ambitions with our actions.

The 2023 UN “gap report” (26) tells us that governments plan to produce around 110% more fossil fuels in 2030 than would be consistent with limiting warming to 1.5°C, and 69% more than would be consistent with 2°C. National carbon-cutting policies are so inadequate that 3°C of heating could be reached this century. Based on existing national pledges, global emissions in 2030 will be only 2% below 2019 levels, rather than the 43% cut required to limit global heating to 1.5°C. To get on track, 22 GtCO_2_ must be cut from currently projected global emissions in 2030. That is 42% of the total and equivalent to the output of the world's five worst polluters: China, US, India, Russia, and Japan.

**Fig. 4.**
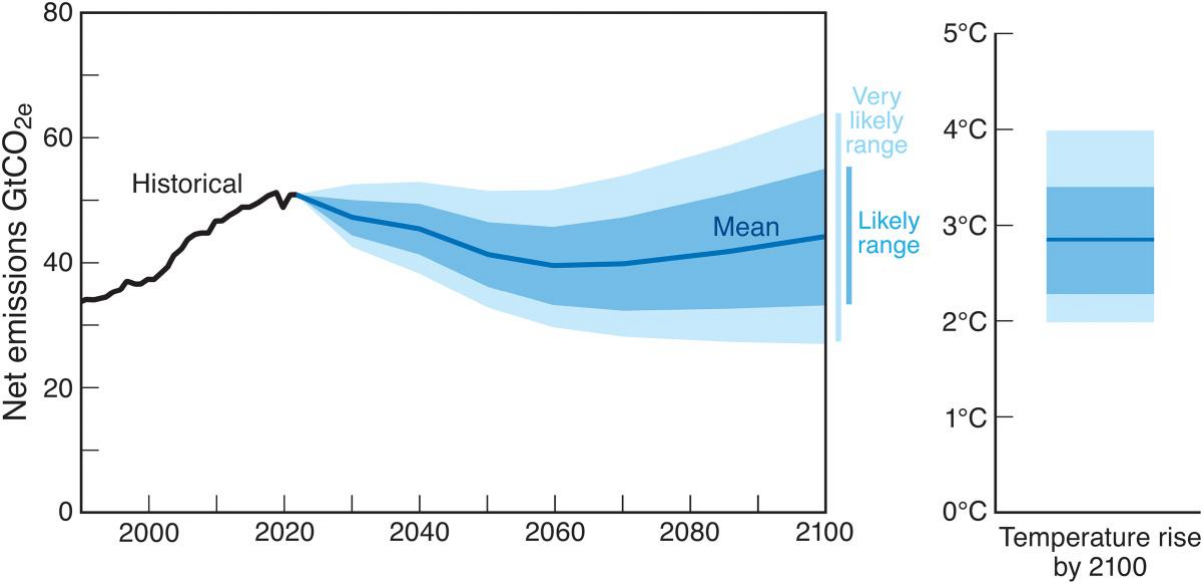
Global GHG emissions and temperature rise. Net emissions including removals (billion metric tons of CO_2_-equivalent). Policy and technological progress over the past 8 years has significantly reduced the global temperature outlook. Models now project very likely temperature increases of 2.0 to 4.0°C by century's end, with a 2.3 to 3.4°C likely range and a mean of 2.8°C. While this is progress from just 8 years ago, it still represents a dire climate future—falling significantly short of the Paris Agreement goal of limiting warming to well below 2°C (204).

The world will need to increase climate spending to around $9 trillion annually by 2030 and to nearly $11 trillion by 2035 to roll out clean sources of energy and prepare for the inevitable impacts of a warming climate during coming decades (201). To limit warming to 1.5°C now requires eliminating emissions shortly after 2040. Although technically feasible, few mainstream scientists believe it is still achievable (202). Instead, analysts predict (203) that global fossil fuel emissions will peak at some point in the next decade, followed not by a decline but a long plateau (204), culminating with end-of-century warming potentially reaching 3°C (Fig. 4).

Although global renewable energy capacity is growing, there is a lack of financing for emerging and developing economies. Redirecting financial resources to lower income nations is crucial. More than 90% of clean-energy investment comes from advanced economies and China, risking new dividing lines in global energy. The biggest shortfalls in clean-energy investment are in emerging and developing economies. More needs to be done by the international community to drive investment in lower income economies, where the private sector has been reluctant to venture. There is ample capital available—evidenced by the nearly $12 trillion allocated for COVID-19 economic relief and the over $1 trillion annually in fossil fuel subsidies, which balloons to $7 trillion with indirect incentives. Reallocating these funds is complex, particularly due to potential impacts on the poorest populations, yet it remains a vital reservoir for investment as the world plans for a sustainable future.

## A new era of reciprocity with nature and among human societies

The purpose of this review is to draw immediate attention to the careless, foolish way that humanity is gambling with the future. Unless things change dramatically, and soon, damage to the natural world will have long-lasting consequences for species and ecosystems, and devastating upheavals for humanity. Although this will particularly affect vulnerable populations, all of humanity faces an unprecedented catastrophe.

There are signs that humanity is awakening to the need for a new system of values that recognize Earth as an island in space with limitations on resource availability. No one is coming to rescue us. Many of the changes that we call for in this essay are consistent with the work of the Intergovernmental Science-Policy Platform on Biodiversity and Ecosystem Services (141) and the UN SDG framework (205). But carbon assimilation in natural systems is decreasing— potentially with significant effects in only decades; planetary-scale biophysical systems such as the Atlantic Meridional Overturning Circulation, the Southern Ocean overturning circulation, atmospheric Hadley circulation, summer sea ice, tropical forests, and others have shifted and are projected to falter. And urbanism, deforestation, consumerism, pollution, disease, social stratification, and extractive agriculture are all on accelerating and expanding trends.

This is a human inflection point that will determine future conditions of life on Earth (206). While transitioning to a carbon-free energy system comes with major societal restructuring, the socioeconomic adjustments needed to rapidly decrease emissions also present opportunities for achieving social and ecological justice, reducing disease, promoting the successful achievement of SDGs, and securing food and water availablity for our children.

We *can* end pollution, improve human health, reign in population growth, and reduce further biophysical risks. Indigenous communities have practiced regenerative ways of managing natural resources by understanding the reciprocal relationship between humans and their natural surroundings. Nature is not a commodity for exploitation, but a living system with its own rights, where humans are life-supported and in turn play a regenerative role. This kinship promotes nature and humanity thriving together.

Under current national plans, global GHG emissions are set to increase 9% by 2030, compared to 2010 levels. Yet the science is clear: emissions must fall by 45% by the end of this decade compared to 2010 levels to meet the goal of limiting global temperature rise to 1.5°C (207). As governments invest in renewable energy sources, there are enormous cobenefits to be gained in terms of disease reduction, social equity, and a growing respect for Earth's rhythms. Yet renewable energy will not address the root problem of ecological overshoot, social justice, or pollution. Policies are needed that end the production of superfluous and luxury commodities, conserve energy at household and societal levels, stabilize global population, and replace the extractive model with one that emphasizes true sustainability so that more natural resources per capita become available and wealth is far more equitably distributed (208).

The shift away from an extractive, resource-driven global economy toward one that values human rights and livelihoods could redefine global economics and offer reasons for optimism. Opportunities to prevent catastrophic levels of heating are being missed due to accelerating consumerism, the false seduction of dubious climate quick fixes, unverifiable “carbon offsets”, exorbitant pollution levels, and growing economic disparity. Halting global ecological decline and addressing the crises of climate change, biodiversity collapse, pollution, pandemics, and human injustice requires a shift in economic structures, human behavior, and above all, values.

Whether the world is considered overpopulated depends on various factors. It is essential to consider not only population numbers but also consumption patterns, resource distribution, and sustainability when discussing this complex issue. Additionally, strategies for addressing concerns related to population growth often involve a combination of policies related to education, healthcare, resource management, and environmental protection. In developing economies, overpopulation is not just about how many people there are but also about how much each person consumes compared to the availability of resources.

High levels of consumption in developed countries contribute to environmental degradation, raising the issue of unequal distribution of resources. While some regions may be densely populated and face resource constraints, others have much lower population densities and abundant resources. Inequities in resource distribution can lead to perceptions of overpopulation but are in reality more closely related to social inequalities, often with deep historical roots related to imperialism and unjust resource extraction.

Humans must become rejuvenators of natural systems (209). We must shift from wealth as a goal, to sustainaiblity as a goal driving our decisons. This includes developing replacements for plastics, adopting regenerative and restorative cultivation and harvesting methods, investing in cradle to grave research and development focused on material reuse, absolute decoupling of the economy from net resource depletion, and establishing conservation goals to conserve 30–50% of Earth's land, freshwater, and oceans (210).

Addressing social inequities based on gender, ethnicity, and income is crucial, and leaders in political, educational, business, and religious organizations must analyze and redress discriminatory practices, historical racism, and unjust distributions of power that hinder communities from adapting to climate change. It is imperative to promote reproductive healthcare, education, poverty eradication, ecological restoration, environmental justice, and reciprocal relationships with nature. Economic development must not come at the cost of destroying Earth.

**Fig. 5.**
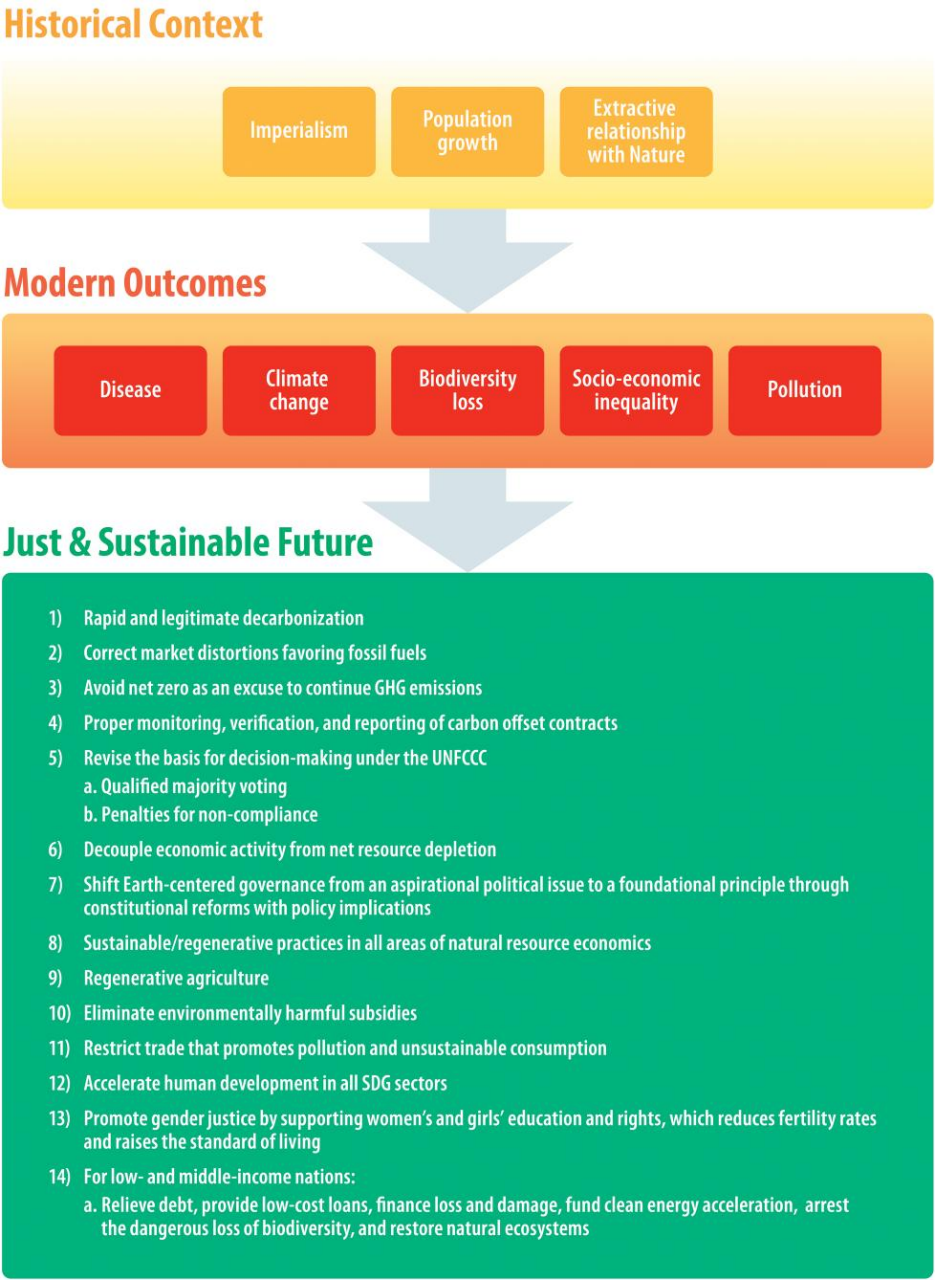
The historical context of imperialism, population growth, and an extractive relationship with nature has led to a series of modern outcomes that put our planet at risk: disease, climate change, biodiversity loss, socioeconomic inequality, and pollution. These risk the stability of human communities. Humanity may achieve a just and sustainable future through global investment in rapid decarbonization, correcting market distortions favoring fossil fuels, avoiding “net zero” as an excuse to continue GHG emissions, proper monitoring and validation of carbon offsets, revising the basis for decision-making under the UNFCCC, decoupling economic activity from net resource depletion, shifting to Earth-centered governance, sustainable/regenerative practices in all areas of natural resource economics, eliminating environmentally harmful subsidies, restrict trade that promotes pollution and unsustainable consumption, accelerate human development in all SDG sectors, promote gender justice by supporting women's and girls’ education and rights which reduces fertility rates and raises the standard of living, and for low- and middle-income nations: relieve debt, provide low-cost loans, finance loss and damage, fund clean-energy acceleration, arrest the dangerous loss of biodiversity, and restore natural ecosystems.

As reported in numerous peer-reviewed studies (211), to reverse the many negative impacts generated by our modern socioeconomic system there must be global investment in (Fig. 5):

1. *Rapid and legitimate decarbonization*, correcting market distortions favoring fossil fuels, avoiding the spurious trap of “net zero” as an excuse to continue polluting the atmosphere (212), and proper monitoring, verification, and reporting of carbon offset contracts.
2. *Revising the basis for decision-making under the UNFCCC.* Decision-making under the UNFCCC should be reorganized by transitioning from unanimous voting to qualified majority voting, enabling decisions to be made with agreement from a defined majority of member nations. To encourage compliance and accountability, penalties such as financial sanctions could be introduced for noncompliance with UNFCCC decisions. These changes would enhance efficiency, enabling prompt action and stronger enforcement of climate-related agreements among member nations.
3. *Building a new era of reciprocity and kinship with nature*, and decoupling economic activity from net resource depletion. We must shift Earth-centered governance from an aspirational political issue to a foundational principle through constitutional reforms with policy implications (213).
4. *Implementing sustainable/regenerative practices* in all areas of natural resource economics including, especially, agriculture.
5. *Eliminating environmentally harmful subsidies* and restricting trade that promotes pollution and unsustainable consumption.
6. *Promote gender justice* by supporting women's and girls' education and rights, which reduces fertility rates and raises the standard of living.
7. *Accelerating human development in all SDG sectors*, especially promoting reproductive healthcare, education, and equity for girls and women.
8. *In low- and middle-income nations*, relieving debt, providing low-cost loans, financing loss and damage, funding clean-energy acceleration, arresting the dangerous loss of biodiversity, and restoring natural ecosystems.

## A cultural shift in values

How do we achieve these goals? The authors call for a global cultural shift in social and economic values. Creating a cultural shift toward regenerative practices in socioeconomic activities is complex and requires a multifaceted approach involving, critically, the leaders of the G20, and all nations, comprehensively engaging programs in the following:

1. *Education in sustainability and equity concepts*: Increasing awareness and understanding of sustainability and equity issues through education at all levels to empower individuals to make more environmentally conscious decisions. Embedding sustainability and equity into educational curricula at all levels can shape future generations’ values and actions. We advocate adoption of the issues discussed in this paper in school curricula, public service announcements, and as a guide to government decision-making.
2. *Policy, legal frameworks, and legislation*: Governments can enact and enforce policies that mandate sustainable practices and ensure social equity, such as progressive environmental regulations, social justice legislation, and economic reforms that prioritize community well-being over individual profit.
3. *Economic incentives*: Shifting the economic focus from growth at any cost to a model that values environmental and social well-being. Aligning economic incentives with sustainable outcomes, such as tax breaks for green businesses, can encourage companies and consumers to adopt better practices.
4. *Cross-sector partnerships*: Facilitating collaboration between the public sector, private sector, civil society, and academia to develop integrated and comprehensive approaches to sustainability and equity.
5. *Community empowerment and inclusion*: Encouraging participatory governance that includes diverse community voices in decision-making processes, particularly those of marginalized and indigenous groups, to ensure that practices are equitable and culturally sensitive.
6. *Corporate responsibility and accountability*: Promoting corporate social responsibility through transparency, fair trade, ethical sourcing, and sustainability reporting.
7. *Incentives for sustainable/equitable behavior*: Channeling investment into the development and deployment of green technologies that enable sustainable production and consumption patterns. Creating economic and social incentives for businesses and individuals to adopt sustainable practices, like subsidies for renewable energy or tax benefits for sustainable/equitable business practices.
8. *Innovation and technology*: Investing in research and development for new technologies can provide more efficient and cleaner alternatives to current practices.
9. *Leadership and commitment*: Encouraging leaders within communities, businesses, and governments to model sustainable and equitable behaviors. Leaders in business, politics, and community groups must commit to sustainability goals and lead by example to inspire others.
10. *Cultural narratives*: Leveraging media, art, and culture to promote stories and images that valorize sustainability and equity, thereby shaping public opinion and cultural values. Changing the cultural narratives around consumption and progress to value sustainability and long-term thinking over immediate gratification or economic growth at any cost.
11. *Global engagement and solidarity*: Participating in international efforts and agreements that aim to address global challenges collectively, ensuring that sustainability and social equity are global priorities.

This systemic transformation requires a shift in collective values, behaviors, and institutional practices to prioritize long-term ecological health and social well-being over immediate gains.

Heads of state must immediately pivot the considerable power of the economy toward restoring a livable planet and an equitable and just socioeconomic system. To achieve a successful future where humanity can thrive, economic values must embrace human equity, health, and welfare, kinship with nature, regenerative resource use, sustainability, and resilience. Emphasizing fairness and inclusivity, these values promote social cohesion and reduce disparities.

Recognizing our interconnectedness with the environment, a focus on sustainability and regenerative resource use ensures the preservation of nature for future generations. Prioritizing health and well-being, societies must invest in healthcare systems, fostering a higher quality of life by building resilience against uncertainties. A new economic paradigm is needed to create a prosperous and harmonious future, meeting the challenges of a rapidly deteriorating world.

## Earth is our lifeboat in the sea of space

**Fig. 6.**
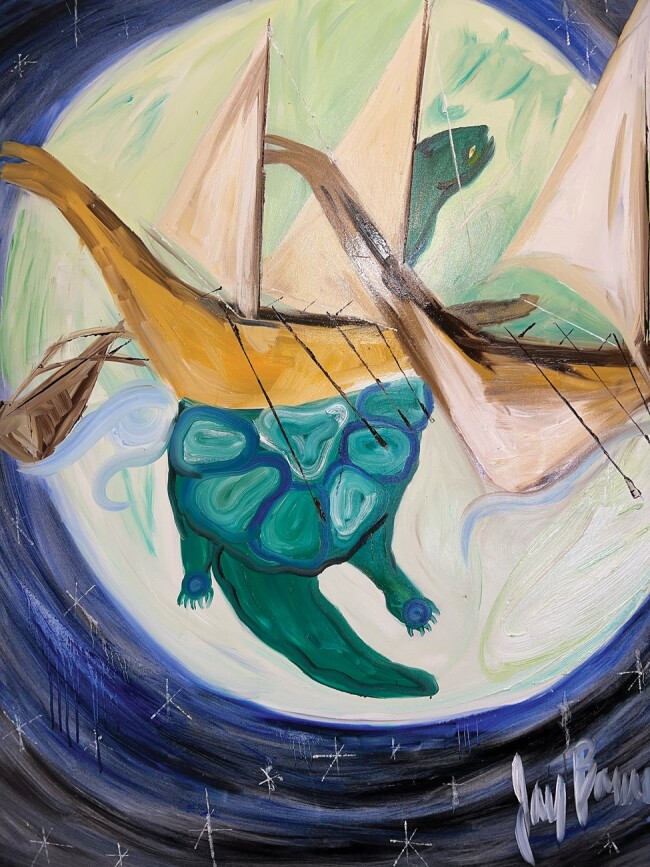
Turtle Island. Original art commissioned for this paper, Jay Bowen (https://jaybowen-art.com/wordpress/).

As succinctly stated by Rees (68), “We are consuming and polluting the biophysical basis of our own existence.” Climate change, biodiversity loss, pollution, disease, and social injustice risk the stability of human communities on Earth (Fig. 6). We must stop treating these issues as isolated challenges, and establish a systemic response based on kinship with nature that recognizes Earth as our lifeboat in the cosmic sea of space.

Coauthor Jay Bowen, Upper Skagit Elder, explained why North American Indigenous Peoples described their North America as “turtle island” (Fig. 6):

“It was not understood why the ancestors had referenced it in this way until the pictures of Earth were seen in 1969 from the Apollo Space Mission. The outline of North America resembled a turtle. We had an understanding of the whole Earth even though we lived on only a tiny piece of it. The ancestors understood global society. We understood that Earth was of one family. This family built and strengthened ties through voyaging to engage in trade, cultural exchange, and discovery.”

There is no guarantee of a just, nourishing, and healthy future for humanity, and hope will not catalyze the change we need. That work must fall upon us, and it is clear from this review that we are past due for, and critically far away from, an appropriate reaction to the global emergency we have created.

## Note

a “Ecological overshoot” is defined as depleting essential ecosystems faster than they can regenerate and polluting the ecosphere beyond nature's assimilative capacity.
